# Congenital skin aplasia on the lower limb in a premature infant with ELBW – case report

**DOI:** 10.1186/s13052-014-0088-0

**Published:** 2014-11-25

**Authors:** Agata Pająk, Anna Szczygieł, Dorota Paluszyńska, Barbara Królak-Olejnik

**Affiliations:** Department and Clinic of Neonatology, Wrocław Medical University, Jan Mikulicz-Radecki University Hospital, Wrocław, Borowska 213 Poland

**Keywords:** Aplasia cutis congenita, Extremely low birth weight neonate

## Abstract

Aplasia cutis congenita (ACC) is usually located on the hairy scalp, on the vertex of the head, but can also occur in other locations, such as limbs, trunk. Congenital skin aplasia on the lower limb is very rare disorder. The exact etiopathogenesis is not known, but intrauterine conditions play a role in its development. ACC visually resembles an ulceration, with a smooth pink surface, which in most cases heals spontaneously. Depending on the wound size and whether signs of inflammation are present, the lesion may require local treatment. In the described case, surgical treatment was carried out because of the extreme prematurity of the infant. The outcome was satisfactory, causing no adverse impact on the child’s development during the infancy.

## Introduction

Aplasia cutis congenita (ACC) occurs with an incidence rate of 1–3 cases per 10 000 live births, regardless of sex or race. In 75% of cases it presents as a scalp lesion on the vertex of the head, but can also appear in other locations, such as limbs or trunk [1]. Over 500 cases have been described worldwide. Congenital skin aplasia on the lower limb is very rare disorder [2]. Eurocat, an institution which gathers statistical data on congenital anomalies worldwide, classifies ACC as a congenital skin disorder and lists a total of 177 cases of this condition registered in Poland in the years 1999–2009. The Polish Registry of Congenital Malformations has gathered data on 168 cases of ACC occurring in the surveyed area between 1998 and 2008; 2 of which were cases with defect located on the lower limb.

Congenital skin aplasia on the limb is a sharply demarcated lesion characterized by the absence of skin, most frequently involving both epidermis and dermis. The lesion resembles an ulceration, with a smooth pink surface where epidermal coverage is either absent or vestigial. Dermis, if present, is thin and lacks skin appendages. The lesion is usually small, measuring roughly 0,5-3 cm in diameter [3]. In about 85% of cases it is an isolated abnormality, but it can, however, converge with other anomalies (e.g. myelomeningocele or corneal opacity) or it can be a symptom in genetic disorders such as Adams-Oliver syndrome or Bart syndrome. It is occasionally associated with epidermolysis bullosa. Histopathological examination is nonspecific [1,3,4].

ACC is classified using the 9-group Frieden classification system, Table 1 [4].

**Table 1:** **ACC classification according to Frieden:** [4]

**Type**	**Short characteristic**
**I**	ACC of the scalp without multiple anomalies. Autosomally dominant or sporadic
**II**	ACC of the scalp with limb reduction anomalies. Autosomally dominant.
**III**	ACC of the scalp associated with epidermal nevi or organ anomalies, associated with corneal opacity and delayed psychomotor development. Sporadic
**IV**	ACC with underlying embryological malformations such as myelomeningocele, spinal cord dystrophy and hemangiomas in the subarachnoid space. ACC in any location, usually the scalp or the abdomen.
**V**	ACC associated with fetus papyraceus and placental infarction. Defect in any location, usually symmetrical and linear. Occasionally accompanied by developmental retardation, nail dystrophy or dystrophy of finger and toe phalanges.
**VI**	ACC associated with epidermolysis bullosa (EB), usually on the limbs. Bullae on the skin and mucosa with accompanying abnormalities of the nails. Inheritance depends on the EB type.
**VII**	ACC located on the limbs, with no bullae and no malformations. Usually located in the shin area and on dorsal surfaces of the hands and feet. Autosomally dominant or recessive
**VIII**	ACC caused by teratogens such as a virus infection (rubella, herpes simplex) or exposure to methimazole. This type usually occurs on the scalp
**IX**	ACC associated with malformations, e.g. chromosome 13 trisomy or epidermal dysplasia.

The etiology of this disorder is unknown. Intrauterine conditions may play a role, e.g. embryological malformations, chromosomal aberrations, epidermal dysplasias, infections (mainly viral ones), placental dysfunction or hypoxia. Cases of familial ACC have been described, as well as the occurrence of ACC in twins. ACC may occur when one of the twin fetuses dies in utero (“fetus papyraceus”), when the umbilical cord becomes wrapped around the fetus and presses against its tissues, in association with epidermolysis bullosa or after exposure to some medications for example methimazole, azathioprine, valproic acid or misoprostol, or to propyrothiouracil [1-8]. A genetic basis is suspected, comprising the deletion of a fragment of the short arm of chromosome 19 (19p13.3), [9] or a mutation with autosomal dominant inheritance, located on the long arms of chromosome 1 and 12 [10].

## Case report

We present the case of a male neonate from a single pregnancy, with a birth weight of 890 g and body length of 39 cm, born after 25 full weeks of gestation by Caesarean section because of impending fetal asphyxia. The child was born in a fair condition, with signs of prematurity and breathing difficulties. It was evaluated at 4/6/6 points on the Apgar score after, respectively, 1/5/10 minutes of life. The umbilical cord was not wrapped around the child at birth. The Caesarean section was performed under local anesthesia. The infant was admitted to the NICU, where he was treated for, among others, respiratory failure, congenital pneumonia and ROP.

The mother was 34 years old, second pregnancy, first birth. The first pregnancy was miscarried in the 14th week. The present pregnancy was complicated by anhydramnios and cervical incompetence. Cervical cerclage was performed in the 18th week of gestation. During the week before birth the mother had elevated markers of inflammation (CRP up to 95,39 mg/l). The day before birth, a swab was taken from the mother’s cervix, from which *Enterococcus faecalis* was cultured. The treatment applied consisted of glucocorticoids (betamethasone) and intravenous antibiotics (ampicillin). The placenta was normal. The family’s medical history was negative for congenital disorders.

**Figure 1:**
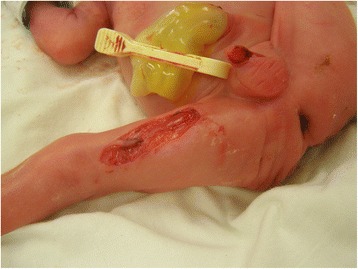
**Aplasia cutis congenita in our patient.**

After birth, the child was diagnosed with a focal skin defect of the right lower limb, sharply demarcated, extending into the subcutaneous tissues, ca. 4 cm long and 1.5 cm wide. This defect was located on the medial aspect of the thigh, involving practically its entire length, Figure 1. On the adjacent skin surface, dried-out vesicles with yellowish contents were present. Microbiological swabs were taken from these lesions, which were found to be sterile. These vesicular lesions disappeared soon after birth. A superficial skin defect on the penis was also present, but it has healed spontaneously in a few days without scar. No other skin defects or nail anomalies were observed.

Initially the thigh ulceration was vividly red, indented below the skin level, dry, no inflammatory. After a surgical consultation, during the first days after birth local treatment was initiated, consisting of antibiotic ointment (mupirocin) and hydrofiber dressings with silver ions. After 4 days of local treatment, the thigh wound was treated surgically and single non-absorbable sutures were placed, adapting the skin. These were left in place for 7 days, with the recommendation to clean the wound daily with a disinfecting agent. After a week the sutures were removed. Due to the wound dehiscence in its central part, measuring about 1 cm, adhesive external sutures were administered. In the following 22 days the wound dehiscence was treated with hydrogel dressings and silver ointment dressings, frequently changed. After the treatment was completed, an uniform linear scar remained on the thigh.

During the entire therapeutic process, full limb function was preserved and no shortening of the limb occurred. No blisters developed on the skin. A histopathological examination was not performed due to the extreme prematurity of the child. During the perinatal and neonatal period, the child’s psychomotoric development proceeded normally for a premature infant. A follow up after 14 months showed a favorable outcome with little scar formation and so functional impairment of the leg.

## Discussion

There has been a case described in the literature of a full-term infant with unilateral absence of skin involving the shin, knee and distal thigh of the right lower limb, extending to the region of the first and second toes. The lesion was ulcerated, with a shiny red surface and visible blood vessels. After two days the presence of granulation tissue was noted within the wound. The defect was locally treated with silver sulfadiazine ointment and compresses. After 3 months of spontaneous healing, a small scar was left on the foot. In the case of this patient, the defect was presumably caused by intrauterine pressure exerted by the umbilical cord which had wrapped itself around the affected limb [5].

Our patient’s skin defect was deeper than the typical ACC lesion, extending below the subcutaneous fat layer, and located in an area which is rarely affected by this disorder. No similar anomalies had been previously noted in our patient’s family.

The neonate came from a single pregnancy, with no information of a twin pregnancy and intrauterine death of one of the fetuses or of amniotic band. The umbilical cord had not wrapped itself around the infant’s body in utero. No placental abnormalities had been noted. Infection by the HSV, VZV, CMV and rubella viruses during gestation was excluded. The mother had not been exposed to any teratogenic substances, such as cocaine, amphetamine, alcohol or other chemicals. The medications administered during pregnancy (betamethasone and antibiotics) are not associated with ACC.

Rajabin *et al.* have described a case of scalp ACC in a 9-year old girl with an accompanying congenital bone defect, syndactyly and brachydactyly of the toes – this patient was diagnosed with Adams-Oliver syndrome. The syndrome described by Bart in 1966 comprises ACC together with nail dystrophy and involvement of the oral mucosa [11]. As mentioned above, our patient had no other congenital anomalies such as cleft palate, umbilical cord hernia, heart defects or digestive tract abnormalities in the shape of pyloric or duodenal stenosis. Thus, the ACC was treated as an isolated anomaly.

On the basis of the clinical picture of the defect and perinatal history, the following diagnosis was made: aplasia cutis congenita of the lower limb of a prematurely born infant with ELBW, type VII in the Frieden classification system.

Very frequently ACC lesions heal spontaneously, leaving a scar, occasionally requiring a modest amount of local treatment. The treatment depends on the wound size and whether signs of inflammation are present. In the case of large head wounds, skin grafting can be considered [12]. During the entire therapeutic process care must be taken to protect the lesion against infection. Due to the size of the wound, premature birth, extremely low birth weight, not yet fully developed skin and difficulties with spontaneous healing, our patient’s defect required surgical intervention.

**Figure 2:**
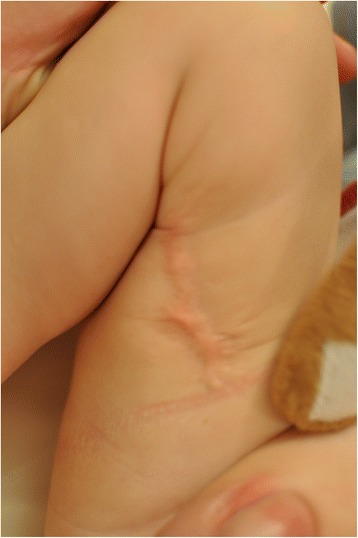
**The same patient after 12 months.**

At present, the 12-month old boy with no skin defect on his right lower limb is developing normally, Figure 2.

## Conclusions

We have described a case of congenital skin aplasia on the medial aspect of the lower right limb in a neonate. This case deserves attention because of the extreme prematurity and extremely low birth weight of the patient, as well as successful outcome.

## Consent

Written informed consent was obtained from the patient’s parent for the publication of this report and accompanying images.
